# Minimally Invasive Dentistry Based on Atraumatic Restorative Treatment to Manage Early Childhood Caries in Rural and Remote Aboriginal Communities: Protocol for a Randomized Controlled Trial

**DOI:** 10.2196/10322

**Published:** 2018-07-25

**Authors:** Peter Arrow, Rob McPhee, David Atkinson, Tamara Mackean, Sanjeewa Kularatna, Utsana Tonmukayakul, David Brennan, David Palmer, Soniya Nanda, Lisa Jamieson

**Affiliations:** ^1^ Western Australia Dental Health Services Research and Evaluation Health Department of Western Australia Perth Australia; ^2^ Kimberley Aboriginal Medical Services Broome Australia; ^3^ Rural Clinical School of Western Australia University of Western Australia Perth Australia; ^4^ Southgate Institute for Health, Society and Equity Flinders University Adelaide Australia; ^5^ Australian Centre for Health Services Innovation School of Public Health and Social Work Queensland University of Technology Brisbane Australia; ^6^ Deakin Population Health SRC Deakin University Melbourne Australia; ^7^ Australian Research Centre for Population Oral Health University of Adelaide Adelaide Australia; ^8^ Community Development Community Development Murdoch University Perth Australia; ^9^ Health Department Western Australia Office of Chief Dental Officer Perth Australia

**Keywords:** cost-effectiveness analysis, early childhood caries, health utility, health-related quality of life

## Abstract

**Background:**

The caries experience of Aboriginal children in Western Australia (WA) and elsewhere in Australia is more than twice that of non-Aboriginal children. Early childhood caries (caries among children <6 years) has a significant impact on the quality of life of children and their caregivers, and its management is demanding and commonly undertaken under general anesthesia. A randomized controlled trial using a minimally invasive dentistry approach based on Atraumatic Restorative Treatment (ART) in metropolitan Perth, WA, has demonstrated a significant reduction in the rate of referral to a dental specialist for dental care among children with early childhood caries, potentially reducing the need for treatment under general anesthesia. The tested approach was clinically successful and was without adverse effects on child dental anxiety. The model of ART-based primary care requires further testing and development if similar outcomes for Aboriginal children in remote and rural settings are to be achieved.

**Objective:**

The study aims to develop, implement, and evaluate a remote primary care model to deliver effective primary dental services, encompassing treatment and preventive services, to Aboriginal preschool children (based on minimally invasive approaches including ART).

**Methods:**

This is a two-arm parallel cluster randomized controlled study in which a test group will be provided with the intervention treatment at the start of the study and a control group will be provided with the intervention treatment 12 months after study commencement (delayed intervention). Participating communities, stratified by size of community (ie, number of children in the sample frame) and baseline caries experience, will be randomly assigned using a computer-generated block randomized list into immediate (test group) or delayed intervention (control group; provided with standard care). Informed consent will be obtained from all participants. Aboriginal research assistants will explain the study to the parents and assist the parents in completing the questionnaires. Participants in the randomized study will be examined at baseline and at 12 months follow-up by a calibrated examiner. Test group participants will subsequently be contacted and appropriate appointments coordinated for treatment. Control group participants will be provided with standard preventive care by the Aboriginal Health Workers and managed for treatment as per standard procedures.

**Results:**

Community consultations have been undertaken and 26 communities have agreed to participate. Fieldwork is in progress to recruit study participants.

**Conclusions:**

The significance of the study lies in its holistic approach to testing the model of care. Clinical evaluations as well as oral health‒related quality of life evaluations will be undertaken. Cost-effectiveness and cost-utility evaluations will assist in the development of policy options for oral health services for rural and remote communities. The elicitation of caregiver perspectives through focus group interviews will supplement the clinical, psychosocial, and cost-utility evaluations and provide a richer evaluation of the intervention.

**Trial Registration:**

Australian New Zealand Clinical Trials Registry ACTRN12616001537448; https://www.anzctr.org.au/Trial/Registration/TrialReview.aspx?id=371735 (Archived by WebCite at http://www.webcitation.org/70UMxndFZ)

**Registered Report Identifier:**

RR1-10.2196/10322

## Introduction

### Background

Dental caries in early childhood, or early childhood caries (ECC), has been shown to have a significant impact on the quality of life of children and their caregivers [1]. These effects include symptoms of pain, functional limitations, psychological dysfunction, parental distress, and financial burden. Also, early life dental caries experience is a strong predictor of dental caries in adulthood [2]. Thus, preventive interventions in the early life course are expected to affect oral health in older age. Therefore, interventions at an early age to manage disease in its early stages and effective preventive measures are needed to maintain function and quality of life and to improve oral health in adulthood.

### Managing Dental Caries in Early Childhood

Oral rehabilitation for dental caries under general anesthesia (GA) of children with ECC has been shown to improve child oral health‒related quality of life (COHRQoL) [3]. However, dental treatment under GA does little to prevent the occurrence of new dental decay in these children and they are often readmitted for dental treatment under GA [4]. Thus, treatment and preventive approaches that can be undertaken in primary dental care settings to reduce the number of preschool children undergoing dental GA are urgently required. There is limited information available on COHRQoL outcomes in children after primary dental care for dental caries that suggests modest improvements in COHRQoL [5]. Therefore, there is a need to evaluate changes in COHRQoL after primary dental care.

Comprehensive care under GA is relatively expensive, for the individual and for the community, and is not without risks, including the potential for long-term adverse neurodevelopmental effects [6-8]. Also, recent reports suggest that oral rehabilitation under GA for children does little to alleviate dental fear or change noncooperative behavior and may in fact heighten child dental fear [9,10].

### Potentially Preventable Hospitalizations

Admissions to a hospital for dental care are classified as potentially preventable with timely and adequate non-hospital care [11]. However, there is a trend of increasing hospital admissions for dental care among children, especially among 0-4-year-olds [12,13]. In Australia, this is occurring in spite of the apparent low dental caries experience among children [14,15]. Admissions to hospital for dental conditions made up more than 20% of total admissions for potentially preventable acute admissions in 2013-14 in Australia, second only behind admissions for urinary tract infections [16]. In a recent report, Western Australia (WA) had the highest, and an increasing, rate of hospital admission for dental treatment of all Australian States and Territories among children. Worryingly, the rate among Australian Aboriginal children was twice that of non-Aboriginal children among the 0-4-year-olds [12]. The cost for hospital admission for dental care for children in WA has been estimated at approximately Aus $9-10 million per year. The mean cost for Indigenous children was significantly higher than the cost of care for non-Indigenous children [6].

### Aboriginal Oral Health

A recent report on the oral health of school children examined within the School Dental Service (SDS) in WA showed that Aboriginal children had nearly twice the decay experience of non-Aboriginal children in both deciduous and permanent teeth, and 1.8 times and 2.4 times the number of carious deciduous and permanent teeth, respectively, after controlling for exposure to community water fluoridation and socioeconomic level [17]. Also, although the rate of admission for hospital-based care has increased for Aboriginal children and is now approaching the rate of non-Indigenous children, it is lower for Aboriginal children in rural and remote areas. This has been attributed to lack of access to care due to costs, availability of services, and a lower proportion of Aboriginal children with dental insurance [6].

In WA, nearly two-thirds of the Aboriginal population lives in rural and remote locations, making access to services challenging [18]. The WA Aboriginal Health and Wellbeing Framework identified oral health among the priorities addressing risk factors, along with development of health services tailored to meet the needs of the Aboriginal people underpinned by evidence, based on quality research [18]. The proposed research will evaluate a model of care, which can be translated into mainstream health service delivery, using a strong randomized controlled study design.

### A “New” Approach to Dental Caries Management in Early Childhood

The minimally invasive dentistry approach to managing dental caries and its potential role in the provision of public dental services has been described in dental literature [19]. The Atraumatic Restorative Treatment (ART) approach, initially developed to assist dental care delivery in underserved communities, where access to electricity and running water may not be readily available, is now increasingly seen to have relevant applications in subpopulations around the world [20]. Whereas the standard care approach would involve the administration of local anethesia and removal of dental caries using rotary instruments, the ART approach principally relies on removing affected dentine using hand instruments alone, usually without the administration of a local anesthetic, and restoration of the prepared cavity with a glass-ionomer cement. ART makes provision of dental treatment in very young children, where cooperation for standard dental care approach may be limited, feasible in a primary care setting. It may also reduce dental anxiety among children, thereby facilitating appropriate future dental attendance behaviors [21].

### Evidence

In WA, dental therapists, through the SDS and working in school-based dental clinics, have been the mainstay of successful publicly provided dental care for 5-17-year-old school children since the early 1970s [22]. A recently completed pilot randomized controlled trial in WA showed that primary care delivered by dental therapists trained in the ART approach, compared to standard care (ie, dentists providing treatment using the drill and local anesthesia), reduced the rate of referral for specialist pediatric dental care of preschool children affected by ECC by 44% [23]. The ART-based approach adopted in the study relied on treating carious teeth by removing affected dentine using hand instruments without the administration of local anesthetic. However, a pragmatic approach to treatment was undertaken in that the use of rotary instruments was permitted where a clinician judged that the child was able to cope with the procedure after a period of acclimatization to dental treatment. This pragmatic approach enabled the undertaking of more invasive procedures, beyond what standard ART approach encompassed, such as pulp therapy of deciduous teeth and, in a few instances, tooth extractions.

The study also included a range of preventive interventions including fluoride varnish application to deciduous molars and noncavitated carious lesions as well as oral health counseling using the motivational interviewing approach. The study delivered a “holistic” package of care that considered the needs of the child in total, including preventive care and appropriate skill development of the parent/caregiver to promote oral health. Also, in that study, some children who were scheduled for care under GA were able to be successfully treated using the ART-based approach. The study showed that the COHRQoL was improved after primary dental care with acceptable clinical outcomes [24,25] without adverse effects on childhood dental anxiety. In addition, the approach was cost saving. Hence, the ART-based approach may provide a successful primary model of care, encompassing both treatment and prevention, for children in rural and remote locations where access to GA may not be advisable or readily available.

### Current Situation

The WA state government is introducing an early childhood preventive program for preschool children (0-4-year-olds) in rural and remote Aboriginal communities by applying fluoride varnish to the primary teeth by Aboriginal health workers trained in the fluoride varnish application. The fluoride varnish program is incrementally being rolled out throughout WA, starting in Kimberley (north-west WA) in 2016. The program is a preventive program, and children requiring dental treatment are referred to the local dental practitioners, either private practitioners or practitioners within the Health Department or Aboriginal Medical Services. While fluoride varnish application has been shown to be efficacious in ECC prevention among Aboriginal children [26], children without adequate access to treatment services may continue to experience untreated disease.

Currently, preschool children (ineligible for SDS care) requiring dental treatment need to source care from private dental practitioners (ie, at their own cost) or, if eligible for subsidized care (ie, liable for co-payments) through government general dental clinics, where restorative care is provided by dentists. Dental therapist and oral health therapists provide mainly dental hygiene services. Subsidized dental care is available to those who are in receipt of certain types of Commonwealth Government benefits (eligibility for the benefits are means tested). The WA government general dental clinics, located mainly in regional cities and major towns, provide clinical care mainly to eligible adults, but children ineligible for SDS and in receipt of specific types of government benefits are able to access care. The location of these clinics in major regional centers means extensive travel for children living in more remote locations to access care. Also, treatment is usually provided using standard care approaches involving the administration of a local anesthetic and using rotary instruments to prepare the cavity.

### Why is this Study Important?

Provision of dental care to preschool children poses significant challenges because of the stage of development and capacity for cooperation of the young child, and treatment is often provided under GA. The issues are multiplied for Aboriginal preschool children in rural and remote locations where access to specialist dental care is severely limited. Alternative approaches to dental treatment of dental decay in preschool children in primary dental care settings that reduce the need for GA is urgently required. This study will test the hypothesis that Aboriginal preschool children in rural or remote locations can be provided with appropriate dental care using the ART-based approach, without the need for specialist care and can potentially avoid the need for GA.

Our study has six major strengths: (1) it will further develop and evaluate the ART-based model of care, which had been successfully tested in a pilot program in an urban setting in WA, led by author PA, (2) we will develop, for the first time, a dental specific child health utility scale, (3) we will measure the change in clinical parameters as well as the changes in child quality of life and health utility with dental treatment, (4) we will measure the impact of dental treatment on child dental anxiety, (5) we will undertake a cost-effectiveness/cost-utility analysis, and (6) we will ascertain the community acceptability of the tested intervention through focus group interviews. The “holistic” evaluation of the intervention including efficacy and effectiveness of the clinical intervention as well as impact on psychosocial parameters and economic evaluation will greatly facilitate research translation. Also, our research team comprise both world-class researchers who have experience working with the Australian Aboriginal population, and leading policy and service delivery experts, thus ensuring the translation of the study findings into applicable policy, practice, and service delivery in rural and remote locations. We will also be working in collaboration with an Indigenous Advisory Committee and significant community members to provide oral health promotion training to significant community members. This will embed oral health promotion within the communities and ensure sustainability of the oral health improvements after the research has been concluded.

The Kimberley region of WA is geographically large, three times the size of the United Kingdom. Almost the entire region of Kimberley (97%) is classified by the Australian Bureau of Statistics as Very Remote with the remaining 3% as Remote. It also has a higher proportion of Aboriginal people than the rest of the state (45% vs 3.6%). There are hundreds of Aboriginal communities dotted throughout the region, and most are very small with few people. The estimated resident population of the region in 2016 was 36,392 (children 0-4 years was 3274). The region also has high levels of socioeconomic disadvantage with the majority of residents (57%) living in areas with the lowest 10% of the Index of Relative Socio-Economic Advantage and Disadvantage score in Australia [27].

### Aims

The principal aim of the proposed study is to develop, implement, and evaluate a remote primary care model to deliver effective primary dental services, encompassing treatment and preventive services, to Aboriginal preschool children (based on minimally invasive approaches including ART). This will be compared with standard care for cost and benefits in terms of improved dental health and reduced childhood dental anxiety. Our specific aims and hypotheses are:

Aim 1. Measure the proportion of children able to be provided with care without the need for dental specialist referral and the increment in dental caries. Hypothesis: The proportion of children successfully managed without specialist referral and without new dental caries will be higher in the test group compared with the control.

Aim 2. Develop a dental-specific health utility scale and measure the change in childhood health utility. Hypothesis: The change in health utility will be greater in the test group compared with the control.

Aim 3. Undertake an economic evaluation of the intervention. Hypothesis: The test intervention will have either less costs with greater/similar health gain or affordable incremental costs for additional unit of health outcomes.

Aim 4. Measure the change in childhood oral health-related quality of life. Hypothesis: The child oral health-related quality of life in the test group will be better than the control

Aim 5. Evaluate the acceptability of the ART-based care through focus group interviews. Hypothesis: The ART-based care will have greater acceptability than the control.

## Methods

### Study Design

We will undertake a two-arm parallel cluster randomized controlled study in which a test group will be provided with the intervention treatment at the start of the study and a control group will be provided the intervention treatment 12 months after study commencement (delayed intervention) in the Kimberley region of WA.

### Ethics

Ethics approval for the study has been provided by the University of Adelaide, Human Research Ethics Committee (HREC) (Ethics approval No. H-2017-015), and the Western Australia Country Health Service HREC (Project Reference #2017/01) and the WA Aboriginal Health Ethics Committee (Project Reference #790).

### Recruitment

We will adopt successful recruitment strategies applied in a wide body of research undertaken by author LJ in her work (unpublished) with Aboriginal communities in South Australia and the Northern Territory. This has included extensive engagement with Aboriginal communities and active community participation in the research process. We will engage with community elders through linkages established by authors DA and RM who both have a long association with Aboriginal communities in the Kimberley region. We will employ local Aboriginal people as research assistants to facilitate with community engagement and participant recruitment guided by a senior project officer based in Kimberley. Participant recruitment will be a two-step process: (1) elicit specific communities to participate, and (2) elicit individual participation. A Senior Research Officer appointed to coordinate the project with the assistance of an Aboriginal research assistant will meet with the Chief Executive Officers of individual Aboriginal communities to explain the proposed study to invite community participation in the study. An Aboriginal Advisory Group with representation from all the Aboriginal Controlled Health Organisations in Kimberley will also be formed to provide appropriate guidance to the research team and assist with information dissemination and participant recruitment. Individual participant recruitment will use active engagement with community members at locations where study participants are likely to gather, such as visits to early childhood learning facilities, community general store, local community women’s functions, and through word-of-mouth dissemination of project information. Parents and guardians will provide signed informed consent after being provided with information about the study and the processes undertaken to protect and preserve data confidentiality.

Parents and children aged 0-4 years, residing in selected communities in the Kimberley region of WA will be eligible. Children with complex medical conditions or developmental syndromes would be excluded. All other children within the scope for age and who consent to participate will be recruited.

Participating communities, stratified by size of community (ie, number of children in the sample frame) and baseline dental caries experience, will be randomly assigned using a computer-generated block randomized list into immediate (test group) or delayed intervention (control group, given standard care) by a central study coordinator. We will select communities for recruitment based on available information on community population from various sources (eg, Community CEOs, the WA Country Health Service, Australian Bureau of Statistics). Communities will also be far enough apart to minimize contamination of test and control (at least 50 km). We will invite participation from communities with at least 100 people to ensure likelihood of recruiting at least 15 children in the age group of interest. We will stratify the communities on size of population and dental caries experience (high vs low; data obtained at baseline examination). Parents of all children of eligible age will be provided with information and consent forms and a questionnaire to complete (Aboriginal research assistants will explain the study to the parents and help parents complete the questionnaires). After receipt of signed consent, participants in the randomized study will be contacted for a baseline clinical examination by a calibrated examiner. After baseline examination, all participants will be contacted by the trial coordinator and appointments arranged for those allocated into the early treatment group. Test group participants will subsequently be contacted and appropriate appointments coordinated to be seen at the local SDS clinics or field clinic settings for treatment. Control group participants will be provided with standard preventive care by the Aboriginal Health Workers and managed for treatment as per standard procedures.

**Figure 1:**
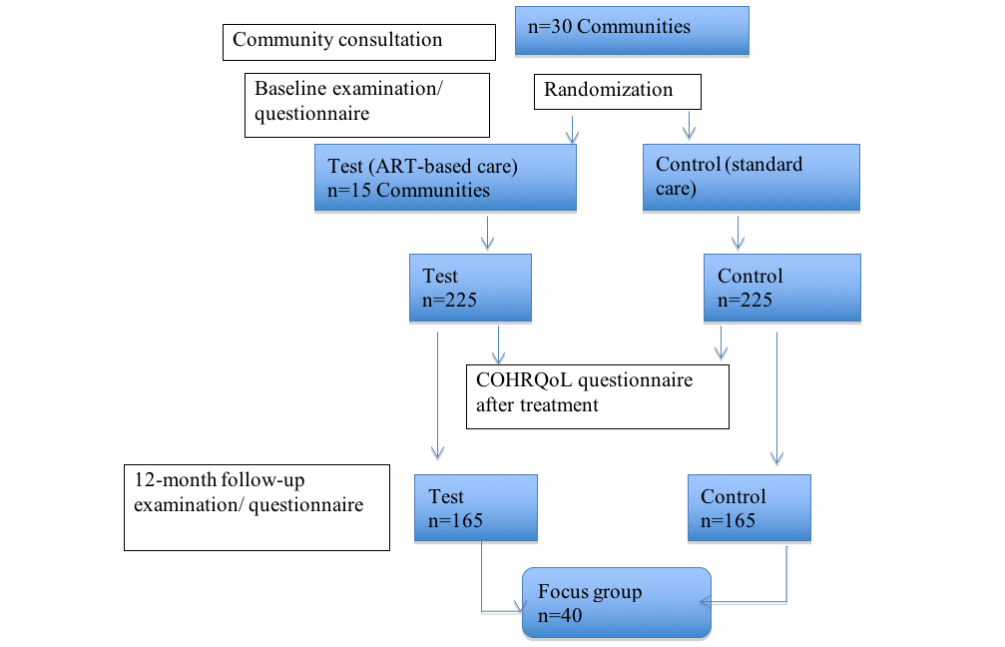
Participant flow chart.

Study participants will be reviewed after 12 months from baseline and will undergo a clinical examination and complete a follow-up questionnaire. Figure 1 shows the study’s participant flow chart.

### Outcomes

Primary outcomes are specialist referral and the increment in dental caries. Secondary outcomes are quality of life and acceptability of the ART-based care.

### Measures

Researchers will help the parents/caregivers complete a baseline questionnaire collecting information on COHRQoL using the Early Childhood Oral Health Impact Scale (ECOHIS) for children ≤6 years old [28]. The scale has been evaluated for Australian children and found to have acceptable reliability and validity [29]. We will also collect parent fear levels using the Index of Dental Fear and Anxiety [30] and child dental fear as reported by the parents as well as self-report by children ≥3 years old using the faces scale [31]. Changes in health utility will be evaluated using the generic Child Health Utility 9D Index (CHU_9D) [32], the Euroqol 5D Youth (EQ-5D-Y), and the new oral health specific utility scale planned to be developed in this study. The ECOHIS has been used successfully among Australian Aboriginal population by author LJ in her National Health and Medical Research Council (NHMRC)‒supported research on pregnant Aboriginal women, while the other measures have been used among disadvantaged populations. We will also evaluate the validity of the questionnaires used among this population.

Participant involvement in the proposed study is shown in Table 1. Both test and control groups will complete the same questionnaire on COHRQoL, child dental fear, the CHU_9D, and the EQ-5D-Y, one month after treatment. The test and control groups will also complete the childhood oral health‒related questionnaire and the CHU_9D and the EQ-5D-Y, at the one-month posttreatment, but anchoring their responses to what their child’s oral health was like before the treatment (“Then-test”) to evaluate the possible effects of response shift [33]. Questionnaires on COHRQoL and parent/child fear and anxiety will again be collected at the 12-month follow-up.

We anticipate the clinical examination to take about 10 minutes, and completion of the questionnaire about 30 minutes. ART-based treatment times will vary depending on the extent of treatment required. Treatments may involve multiple appointments, usually about 20 minutes at each appointment. A sample of study participants will be invited to focus group interviews, which will take approximately 1 hour per group.

All participants will also be clinically examined at the 12-month follow-up, by a “blind” calibrated examiner to evaluate their oral health status. Effectiveness will be assessed by the number of teeth treated, dental caries increment, and changes in quality of life. The quality of the treatment provided will be assessed by blind calibrated examiners at follow-up, 12 months after treatment, using established criteria for determining ART restorations [34]. Clinical status of the teeth will be assessed using International Caries Detection and Assessment System-II criteria, which span the continuum from sound to extensive decay [35].

### Economic Evaluations

#### Cost-Effectiveness Analysis

Cost-effectiveness of the intervention will be estimated using standard approaches [36] and will be from a health care provider perspective. Effectiveness measures will be the number of children managed in primary care without need for specialist referral, changes in COHRQoL, the numbers and types of treatments provided, and dental caries increments. The economic evaluation will compare any incremental costs of the intervention (ie, costs accrued in the intervention arm compared to those in the control arm) to the full list of incremental primary and secondary outcome endpoints, all expressed in their natural units of measurement. Costs will be measured from activity data with pathway analysis to fully specify all activities in both intervention and control arms. The resource use and dental services utilization will be obtained from research team records and intervention provider records. Measured resource use will be valued using both existing estimates of the costs of each unit of the resource use from market prices and the Dental Benefit Schedule fee rates for nonspecialist and specialist attendances. Standard discounting will be applied to both cost and outcomes. Uncertainty in the cost and outcome data will be subjected to sensitivity analyses.

#### Cost Utility Analysis

Cost-utility analysis will be undertaken using scores derived from the CHU_9D [37,38], the EQ-5D-Y, and the new oral health specific utility scale developed in this study. The use of scores from the CHU_9D as outcome measures in child dental health has been suggested [39]. However, in a preliminary evaluation as an outcome measure it was found not to be sensitive to changes in childhood oral health [40], while the use of the adult version of the EQ-5D has been suggested as being able to differentiate oral health states [41]. Hence, we will further test the usefulness of the CHU-9D as an outcome measure for childhood oral health and seek to develop an oral health specific multi-attribute utility instrument within this project.

**Table 1:** Participant involvement in the proposed study.

Timepoint	Intervention group (test)	Delayed intervention group (control)
Baseline	Questionnaire: ECOHIS, CHU_9D, EQ-5D-Y, Dental Utility Scale, dental fear and anxiety of parent and child; Clinical assessment of children; ART-based care	Questionnaire: ECOHIS, CHU_9D, EQ-5D-Y, Dental Utility Scale, dental fear and anxiety of parent and child; clinical assessment of children; standard care
1 month after baseline treatment	Questionnaire: ECOHIS, CHU_9D, EQ-5D-Y, Dental Utility Scale; child dental fear and anxiety	Questionnaire: ECOHIS, CHU_9D, EQ-5D-Y, Dental Utility Scale; child dental fear and anxiety
6 months after baseline treatment	ART-based care; focus group interviews	Standard care; focus group interviews
12 months after baseline treatment	Questionnaire: ECOHIS, CHU_9D, EQ-5D-Y, Dental Utility Scale; child dental fear and anxiety; clinical assessment	Questionnaire: ECOHIS, CHU_9D, EQ-5D-Y, Dental Utility Scale; child dental fear and anxiety; clinical assessment; ART-based care

### Dental Specific Multi-Attribute Utility Instrument and a New Dental Utility Scale

We will use the available dataset of ECOHIS from the 250 respondents in the recently completed WA pilot study [29]. Although valid and widely used, available quality of life instruments in oral health cannot be used to measure quality-adjusted life years (QALYs). We will use the methodology described by Brazier and Rowen et al [42,43] to guide the development of the new oral health specific preference-based instrument. This includes a six-stage approach: establish dimensionality (Stage 1), eliminate and select items per dimension (Stage 2), explore item-level reduction (Stage 3), validate instruments (Stage 4), apply the new instrument to elicit health state values for a sample of health states described (Stage 5), and analyze the model results to produce utility values for all health states (Stage 6). The new Dental Utility Scale will then be developed.

This will be the first multi-attribute utility instrument in oral health conditions as well as the first validated multi-attribute utility instrument in this age group. This new instrument and its scale will be used to calculate QALYs for the cost-utility analysis.

### Treatment Procedures

Test children will be provided with care by dental therapists previously trained in and using the ART approach. Treatment will be undertaken at SDS clinics or field settings, using portable equipment. Restorative treatments will be provided using hand instruments principally, without the use of local anesthesia with the cavity prepared and subsequently restored with a glass-ionomer cement. Where extractions are required, the procedure will be undertaken using standard care approaches. All children will also be provided with preventive fluoride varnish applications at treatment and reviewed at 6-month follow-ups. All treatment will be recorded in patient clinic records. At the 6-month reviews, participants will be provided with preventive fluoride varnish and any other necessary care. Children unable to be provided with care will be referred for specialist care. There will be no direct costs incurred by the participants for the primary care.

Control children will be provided with standard care as part of the fluoride varnish program. Children found to require dental treatment will be referred for care through the prevailing care pathway, that is, government dental services or local private practitioners.

In order to ensure all participants are offered the opportunity to access dental treatment, and in keeping with the delayed treatment intervention design, control participants will be offered treatment using the test treatment approach after the 12-month follow-up.

### Focus Group

A sample of parents from the randomized test and control groups will be invited to participate in focus group interviews to elicit their perspectives on the care provided to their child using the various approaches (including those referred for GA care). Children will not be involved in the focus group interviews due to their young age. Previous experience with focus group interviews indicated that a sample of about 20 from each arm of the study is sufficient to reach saturation.

The focus group questions will be based on the following:

1. What were some of the positive aspects of dental care your child experienced?
2. What were some of the negative aspects of the dental care your child received?
3. Can you give some examples of what you think could have been done/implemented better during your child’s treatment?
4. Can you name some aspects of the setting/location/process that you think could have been improved?
5. Can you identify any changes to your oral health knowledge since the research began?

### Sample Size

A recently completed pilot study in WA, which compared ART-based care against standard care, reported a nine-fold difference in the proportion of children referred for specialist pediatric dentist care (5% vs 49%). A conservative difference in effect size of 2.5 was assumed to estimate the sample size (10% vs 25%). The intracluster correlation was estimated from the dental caries experience of Aboriginal children participating in a NHMRC Project Grant (1010758)‒funded cluster randomized trial (.05). Using these parameters, with 15 clusters available in each arm of the trial, the estimated sample size required, at 80% power and alpha of .05, was 165 in each arm of the trial with 11 children per cluster. Allowing for loss to follow-up of 25%, the estimated sample size is 220 per arm of the study or 15 children per cluster. The recently completed pilot trial in WA, undertaken by author PA, achieved 90% retention of study participants after 12 months, while an oral health promotion intervention undertaken by author LJ among an Aboriginal population achieved 80% retention of study participants. The estimated sample size will have sufficient power to detect a 33% difference in mean ECOHIS at 90% power and alpha of .05.

The study timeline is shown in Table 2. It is expected that community engagement will take the bulk of the first year in recognition of the challenges of undertaking research in northwestern WA, which is as far as Sydney is from Perth (about 3000 km). Participant recruitment and treatment provision is similarly expected to take up to 2 years because of constraints of locations of communities and weather conditions.

**Table 2:** Study timeline, indicated by actions taken per quarter.

Action	Year 1				Year 2				Year 3				Year 4				Year 5			
	1	2	3	4	1	2	3	4	1	2	3	4	1	2	3	4	1	2	3	4
Community engagement, staff recruitment, training, ethics approval, printing	✓	✓	✓	✓																
Participant recruitment, baseline, examinations, randomization, commence treatment, baseline data entry			✓	✓	✓	✓	✓	✓	✓	✓										
Follow-up examinations, focus group interviews, delayed intervention group treatments							✓	✓	✓	✓	✓	✓	✓	✓	✓					
Data entry, data clean-up, data analyses									✓	✓	✓	✓	✓	✓	✓	✓	✓			
Report preparation, community presentation and feedback																	✓	✓	✓	✓

### Data Analysis

Data will be analyzed on an intention-to-treat (ie, participants analyzed on the basis of their group allocation regardless of whether they received the intended treatment), and per protocol basis (participants analyzed on the basis of their group allocation and receiving the intended treatment). Descriptive statistics will be presented and baseline variables will be compared between groups to test for fairness with respect to the randomization. Aim one (primary outcome) will be tested using test of proportions and logistic regression to control for potentially confounding factors. Changes in health utility and COHRQoL (secondary outcomes) will be tested using paired (within group) and unpaired (between groups) and parametric and nonparametric tests as appropriate, and multivariate analysis using linear regression for continuous variables and Poisson regression for count variables to control for possible intergroup imbalances. Responsiveness of the COHRQoL scale will be determined by calculation of effect sizes for the scale overall and specific domains. Statistical significance will be set at alpha=.05. All analyses will take into account the cluster design and will incorporate multilevel analyses where indicated. Multiple imputation of missing data will be further undertaken to evaluate its impact on the primary and secondary outcomes.

### Qualitative Analysis

NVivo9 computer software will be used to code the transcripts from the focus groups. Emergent themes from the focus groups will then be explored (n=20 from each arm). Transparent validation of emergent themes and content will be performed using 2 coders. Thematic analysis will be performed as such an approach allows for contextual differences between perceptions, experiences, and belief to be developed and explored.

Incremental cost-effectiveness ratio—the ratio of incremental costs to incremental outcome between the test and control group—will be calculated. Incremental outcomes for the cost-effectiveness analysis include differences in number of children treated in primary care settings without the need for specialist pediatric dental referral, the differences in COHRQoL between and within groups, the differences in number and type of treatment provided, re-treatment, and antibiotics for dental infections over the 12-month period. Incremental outcome for cost-utility analysis refers to difference in health utility scale.

### Data Quality Control

Hard copy data will be entered electronically into a database software, and data will be checked at entry. The database will have data validation parameters incorporated to alert for any values that are outside of permissible values. Participants will be contacted to clarify and amend ambiguous or confusing responses. Data cleaning will be undertaken with 2 people, one to scan the data entry and the other to verify from the hard copy.

## Results

Community consultations have been undertaken, and 26 communities have agreed to participate. Fieldwork is in progress to recruit study participants.

## Discussion

### Principal Considerations

Closing the gap in Aboriginal child health is a national priority. A lack of access to dental services by rural and remote Aboriginal communities has been highlighted. The outcomes of the proposed study will address multiple goals of the NHMRC 2013-15 strategic plan, primarily to “improve the health of Aboriginal peoples and Torres Strait Islanders through the support of health research and its translation”. The research team, comprising established oral health researchers and child health researchers working in Aboriginal health, community development, health economists, dental practitioners, and oral health policy makers, will ensure that the findings of the study can be readily translated into policy and practice. Second, the research is driven by provision of care at the primary care level and will test the capacity of the intervention to reduce the need for tertiary care at hospital for a condition that is essentially a preventable hospital admission and to reduce health inequalities. Third, our research proposal supports the NHMRC goal of “healthy start for healthy life” by engaging with Aboriginal families in the provision of dental care by offering treatment and preventive services.

The outcomes will have a direct impact on the COHRQoL for the study participants because all participants in the treatment arms will be provided with an opportunity to receive dental treatment. The research will also have flow-on effects through the demonstration of a model of care with potential applications in other settings throughout Australia, such as aged care facilities and nursing homes and among population with disabilities/special needs. The study also addresses the oral health needs of priority populations identified in the Australian National Oral Health Plan, 2015-2024, specifically, Aboriginal and Torres Strait Islander people. It will also have impacts for other priority populations identified in the National Oral Health Plan, namely, people who are socially disadvantaged or on low incomes, and people living in regional and remote areas.

We will further undertake oral health promotion and community development to ensure sustainability of the oral health promotion activities by engagement with the Aboriginal Communities and community champions. Furthermore, the development of a condition specific health utility scale will be a major advancement in enabling economic evaluation of oral health care programs for young children using a preference-based measure.

We will also employ Aboriginal research assistants who will be trained in dental clinic assisting as well as research processes. They will also participate in the oral health promotion activities and in the process will be trained to undertake community oral health promotion activities, which will add to capability development within Aboriginal communities. We will also disseminate the study findings to the participating communities by holding community forums as well as ad hoc sit-down chats to present study findings and will meet with the Chief Executive Officers of the Aboriginal Communities to report on the study findings as well as provide them with a written report.

### Conclusion

The significance of this study lies in its holistic approach to testing the model of care. Clinical evaluations as well as oral health‒related quality of life evaluations will be undertaken. Cost-effectiveness and cost-utility evaluations will assist in the development of policy options for oral health services for rural and remote communities. The elicitation of caregiver perspectives through focus group interviews will supplement the clinical, psychosocial, and cost-utility evaluations and provide a richer evaluation of the intervention.

## Abbreviations

ART: atraumatic restorative treatment
CHU_9D: Child Health Utility 9D Index
COHRQoL: child oral health–related quality of life
ECC: early childhood caries
ECOHIS: Early Childhood Oral Health Impact Scale
EQ-5D-Y: Euroqol 5D Youth
GA: general anesthesia
HREC: human research ethics committee
NHMRC: National Health and Medical Research Council
QALYs: quality-adjusted life years
SDS: School Dental Service
WA: Western Australia
